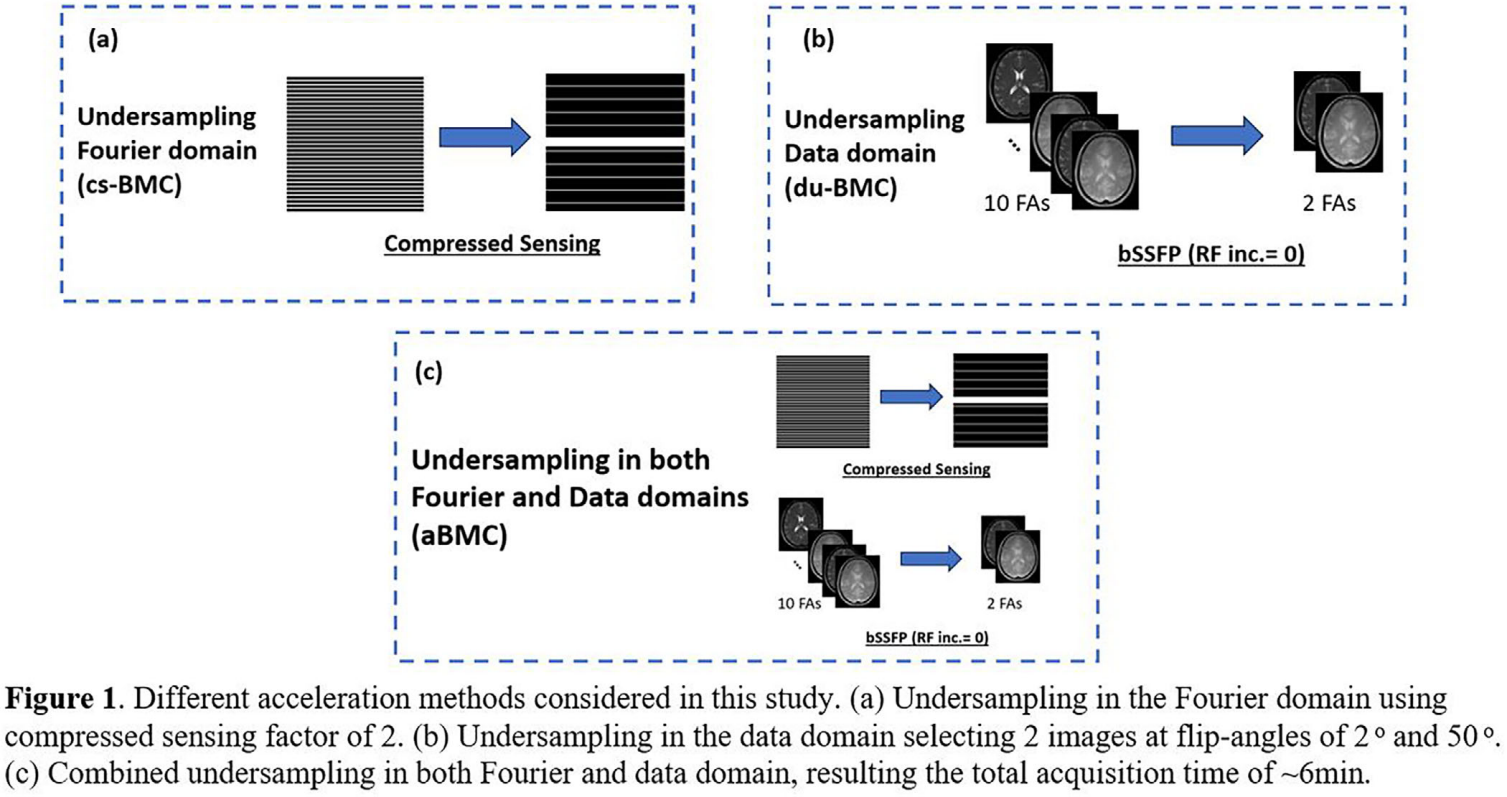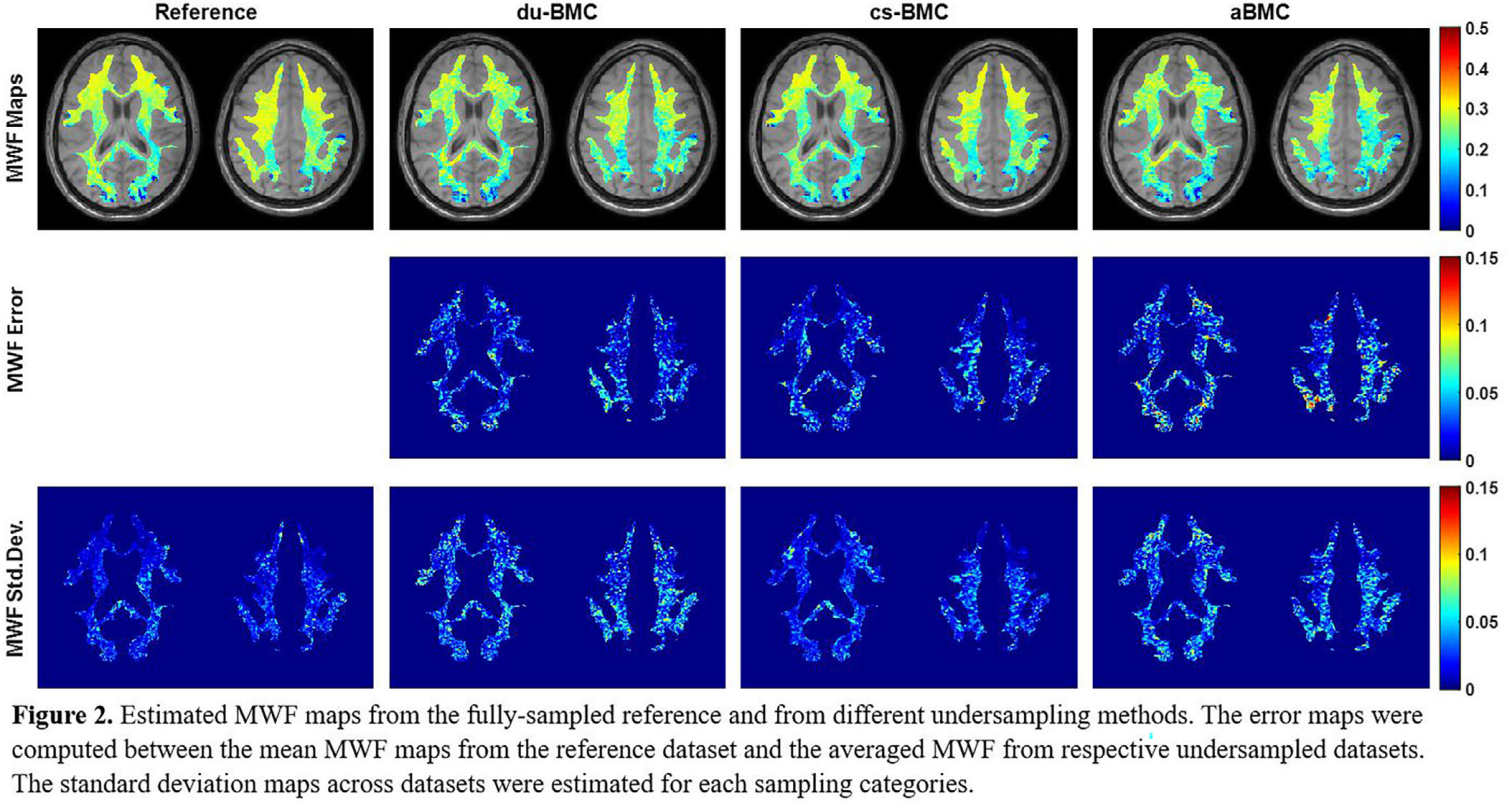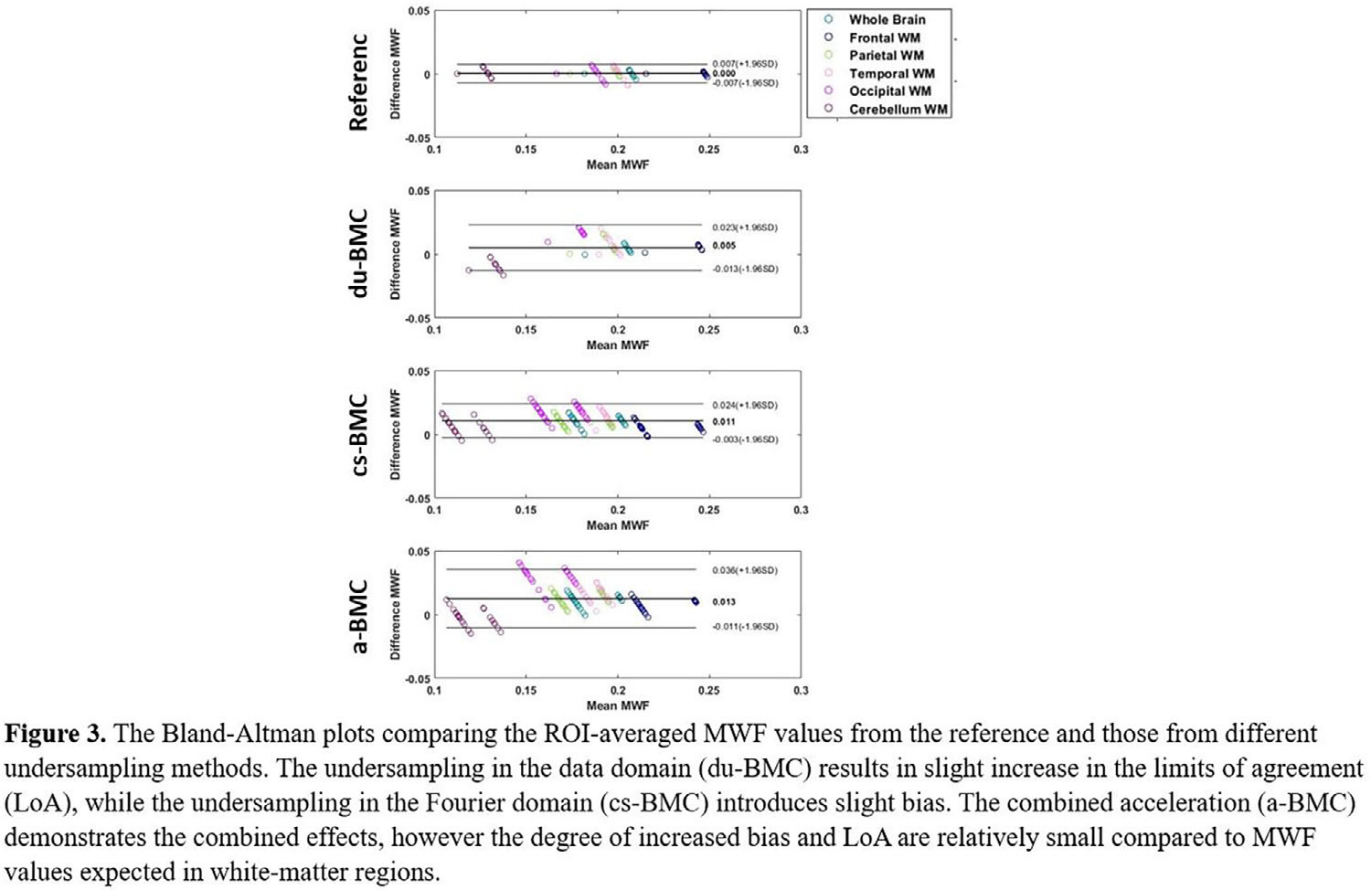# Myelin water imaging with clinically feasible scan time: the accelerated BMC‐mcDESPOT (aBMC‐mcDESPOT) method

**DOI:** 10.1002/alz.093655

**Published:** 2025-01-09

**Authors:** Jonghyun Bae, Zhaoyuan Gong, Alex Guo, John P Laporte, Mary E Faulkner, Mustapha Bouhrara

**Affiliations:** ^1^ National Institute on Aging, Baltimore, MD USA; ^2^ Laboratory of Clinical Investigation, National Institute on Aging, Intramural Research Program, Baltimore, MD USA

## Abstract

**Background:**

In 2016, we introduced the Bayesian Monte Carlo analysis of multicomponent‐driven equilibrium observation of T1 and T2 (BMC‐mcDESPOT) MRI method for myelin water fraction (MWF) mapping, a surrogate of myelin content. While BMC‐mcDESPOT has been extensively applied to study brain aging, dementias, and risk factors influencing myelination, it still requires a lengthy acquisition time (∼17 min) which hampers its integration in clinical studies and trials. In this study, we aim to accelerate the BMC‐mcDESPOT method for whole brain, high‐resolution, MWF mapping within clinically feasible scan time of ∼6 min.

**Method:**

Data Acquisition Reference data (ref‐BMC, no‐acceleration): Two subjects underwent the BMC‐mcDESPOT protocol consisting of SPGR and bSSFP images at 10 different FAs. For assessing reproducibility, three sets of fully‐sampled datasets were collected without any under‐sampling (total acquisition time = 17 min). Compressed sensing acceleration (cs‐BMC): Three sets of accelerated scans were acquired using compressed sensing (CS) with an acceleration factor of ∼2 (9 min) (Figure 1(a)). Image domain acceleration (du‐BMC): The undersampling in the data domain was retrospectively performed on the bSSFP images by selecting only 2 optimal images instead of the full 10 images (12 min) (Figure 1(b)). Combined accelerations (aBMC): The above two acceleration methods were combined and resulted in an acquisition time of ∼6min. (Figure 1(c)) Data Processing We estimated MWF maps from the different undersampling methods described above. The accuracy was evaluated between the MWF from the reference and those from each undersampled dataset. The reproducibility was measured with the coefficient of variations (CoV) across different measurements for each sampling method. We also performed Bland‐Altman analysis to quantify bias and limits of agreement (LOA).

**Result:**

Figure 2 shows a representative slice of MWF estimates from different sampling methods. The different acceleration methods showed similar regional patterns, with relatively low errors (du‐BMC/cs‐BMC/aBMC = 10.8/9.8/13.7%) and CoV (ref‐BMC/du‐BMC/cs‐BMC/aBMC = 9.0/14.3/10.7/15.3%). While a slight bias in the derived MWF values was observed (Fig.3), the LOA for aBMC was under 5% in white matter.

**Conclusion:**

aBMC‐mcDESPOT is a practical MRI method for accurate determination of MWF within 6 min, offering unique opportunities to further integrate myelin imaging in clinical studies and trials.